# Glutamate Carboxypeptidase II in Aging Rat Prefrontal Cortex Impairs Working Memory Performance

**DOI:** 10.3389/fnagi.2021.760270

**Published:** 2021-11-15

**Authors:** Dibyadeep Datta, Shannon N. Leslie, Elizabeth Woo, Nishita Amancharla, Ayah Elmansy, Miguel Lepe, Adam P. Mecca, Barbara S. Slusher, Angus C. Nairn, Amy F. T. Arnsten

**Affiliations:** ^1^Department of Neuroscience, Yale University School of Medicine, New Haven, CT, United States; ^2^Department of Psychiatry, Yale University School of Medicine, New Haven, CT, United States; ^3^Department of Neurology and Johns Hopkins Drug Discovery, Johns Hopkins School of Medicine, Baltimore, MD, United States

**Keywords:** NAALADase, FOLH1, mGluR3, GRM3, inflammation, 2-MPPA

## Abstract

Glutamate carboxypeptidase II (GCPII) expression in brain is increased by inflammation, and reduces NAAG (N-acetyl aspartyl glutamate) stimulation of mGluR3 signaling. Genetic insults in this signaling cascade are increasingly linked to cognitive disorders in humans, where increased GCPII and or decreased NAAG-mGluR3 are associated with impaired prefrontal cortical (PFC) activation and cognitive impairment. As aging is associated with increased inflammation and PFC cognitive deficits, the current study examined GCPII and mGluR3 expression in the aging rat medial PFC, and tested whether GCPII inhibition with 2-(3-mercaptopropyl) pentanedioic acid (2-MPPA) would improve working memory performance. We found that GCPII protein was expressed on astrocytes and some microglia as expected from previous studies, but was also prominently expressed on neurons, and showed increased levels with advancing age. Systemic administration of the GCPII inhibitor, 2-MPPA, improved working memory performance in young and aged rats, and also improved performance after local infusion into the medial PFC. As GCPII inhibitors are well-tolerated, they may provide an important new direction for treatment of cognitive disorders associated with aging and/or inflammation.

## Introduction

The enzyme glutamate carboxypeptidase II (GCPII, encoded by the *FOLH1* gene) is of growing interest to neuroscientists, as increased GCPII expression is associated with impaired cognitive function in humans (Zink et al., 2020). GCPII is a transmembrane zinc metallopeptidase that catabolizes NAAG (N-acetylaspartylglutamate), the endogenous ligand for mGluR3 (glutamate metabotropic receptor type 3) (Coyle, 1997; Wroblewska et al., 1997; Neale et al., 2000). Human subjects carrying a gain-of-function mutation in the *FOLH1* gene express higher levels of GCPII resulting in decreased levels of NAAG in the brain, as well as inefficient activation of the dorsolateral prefrontal cortex (dlPFC) during working memory, and lower IQ (Zink et al., 2020). Genetic alterations in mGluR3 (*GRM3*) are also a replicated risk factor for schizophrenia (Saini et al., 2017). Thus, understanding the role of GCPII-NAAG-mGluR3 signaling in PFC functioning has direct clinical relevance. As GCPII expression is increased by inflammation (Neale et al., 2005; Cao et al., 2015, 2016; Zhang Z. et al., 2016), and during drug withdrawal (Hicks et al., 2017), GCPII inhibition has therapeutic potential for a range of cognitive disorders associated with inflammatory and dysregulated etiologies.

Although NAAG is the third most prevalent neurotransmitter in the brain (Neale et al., 2011), the roles of NAAG-mGluR3 signaling have been less studied than other glutamatergic mechanisms. It is now well-established that NAAG is co-released with glutamate, but unlike glutamate, is selective for mGluR3 and does not bind other glutamate receptors, and commonly leads to G_*i*_ inhibition of cAMP signaling (Neale, 2011). GCPII catabolizes NAAG, and thus reduces endogenous mGluR3 signaling (Neale et al., 2011). GCPII is also highly expressed outside of the nervous system in several tissues, including the prostate, where it can be synthesized and released by epithelial cells and endocytosed into surrounding cells (Bařinka et al., 2012). In brain, GCPII mRNA is concentrated in astrocytes (Berger et al., 1999). Physiological recordings from monkeys performing a working memory task have shown that local administration of a GCPII inhibitor enhanced task-related neuronal firing (Jin et al., 2018), suggesting that GCPII inhibition may have therapeutic value, especially in conditions associated with neuroinflammation.

Advancing age is associated with increased inflammation (Simen et al., 2011), and aged rats are known to have increased complement C1q expression (Datta et al., 2020) and dysregulated cAMP-PKA signaling in the prelimbic medial PFC (PL mPFC) associated with impaired working memory performance (Ramos et al., 2003; Carlyle et al., 2014; Leslie et al., 2020). However, it is not known whether there is increased GCPII expression in the aging rat mPFC, and whether GCPII has detrimental effects on PFC cognitive function.

The current study examined GCPII expression in the rat mPFC, examining whether GCPII protein expression increased with age, and whether local and/or systemic inhibition of GCPII with the most brain penetrant available compound, 2-(3-mercaptopropyl) pentanedioic acid (2-MPPA) (Van Der Post et al., 2005; Vornov et al., 2013), would improve working memory performance in young adult and aged rats. We found that GCPII was expressed not only on astrocytes as expected, but also on neurons, and that GCPII levels increased with age. GCPII appears to be detrimental to PFC cognitive functioning, as either local or systemic administration of 2-MPPA improved working memory performance.

## Materials and Methods

All methods followed NIH and USDA guidelines and were approved by the Yale IACUC.

### Subjects

A total of 55 adult male (3–31 months) Sprague-Dawley rats (Envigo; Indianapolis, Indiana) were used in the present study. Rats in behavioral studies were single-housed under a 12 h light/dark cycle, and maintained on a diet of Purina rat chow. The immunohistochemical experiments utilized 3 young (3 months) and 3 aged (27–29 months) rats; the biochemical experiments utilized 20 rats, 10 young (average of 3.5 months) and 10 aged (average of 27.8 months) Tissue from these animals has been previously analyzed in Datta et al. (2020) (C1qA) and Leslie et al. (2020) (PDE4D, pS214-tau). The assessment of 2-MPPA on working memory performance utilized a total of 29 rats: 9 young (3–4 months) and 12 aged (24–31 months) for systemic studies, and 8 adult rats (∼12 months) for the infusion experiments. Cognitively characterized rats were fed a regulated diet (∼16 g/day) immediately after behavioral testing to maintain motivation for task performance with water available *ad libitum*. These rats showed normal growth curves.

### Antibodies

For mGluR3 immunohistochemistry, a well-characterized rabbit polyclonal antibody raised against a synthetic peptide corresponding to the N-terminal (extracellular) domain of human mGluR3, and purified by peptide immunogen affinity column, was obtained commercially (G1545, Sigma-Aldrich) for use in this study. The immunizing peptide demonstrates 100% homology with the rat gene. This antibody has been extensively tested, and shown by others to specifically recognize mGluR3 in human brain using both immunohistochemical and immunocytochemical approaches (G1545, Sigma-Aldrich). The specificity of this antibody has also been confirmed in *in vitro* assays and by western blotting using small interfering RNA for genetic suppression of mGluR3 expression in rodent hypothalamic and cortical cultures (Park et al., 2011; Wang et al., 2012). This antibody has been validated by our own group in monkey dlPFC (Jin et al., 2018). For GCPII immunohistochemistry, we used the mouse PSMA/FOLH1/NAALADase I/GCPII antibody raised against Lys44-Ala750 which contains the extracellular domain of GCPII (MAB4234; R&D Systems). The antibody recognizes a very specific band for GCPII by western blotting with no other non-specific bands. The recognition of PSMA/FOLH1/GCPII was confirmed using antigen-down ELISA (R&D Systems). In direct ELISA’s, the antibody shows less than 10% cross-reactivity with rhNAALADase-like 2 and no cross-reactivity with rhNAALADase-like 1, rhNAALADase-like 3, rmNAALADase I, or rmNAALAase-like 2. Our own histology methodological controls are summarized below. For mGluR3 and GCPII biochemistry, antibodies used in this study include GAPDH (Millipore CB1001–500, 1:10,000), mGluR3 (Abcam ab166608, 1:1,000), GCPII (VWR 10092-886, 1:500).

### Immunohistochemistry

Tissue preparation and imaging: Animals were anesthetized with Nembutal (50 mg/mL, i.p.) and perfused transcardially with 4% paraformaldehyde (PFA) in 0.1 M phosphate buffer (PB; pH7.4). After perfusion, brains were removed from the skull and immersed in 4% PFA overnight at 4°C. Coronal 60 μm-thick sections were then cut on a Vibratome (Leica V1000) and collected in 0.1 ml PB. Sections were collected over the full rostrocaudal extent of the mPFC. The sections were cryoprotected through increasing concentrations of sucrose solution (10, 20, and 30% each for 2 h, then 30% overnight), cooled rapidly using liquid nitrogen and stored at –80°C. In order to enable penetration of immunoreagents, all sections went through 3 freeze-thaw cycles in liquid nitrogen. Following thawing, sections of the mPFC were transferred for 1 h to Tris-buffered saline (TBS) containing 5% bovine serum albumin, plus 0.3% Triton X-100 to block non-specific reactivity and enhance permeabilization, respectively, and incubated with primary antibody in TBS for 48 h at 4°C.

For mGluR3 immunohistochemistry, we used the mGluR3-specific rabbit antibody at 1:200 dilution. For GCPII immunohistochemistry, we used the mouse PSMA/FOLH1/NAALADase I/GCPII antibody at 1:100 dilution. The tissue sections were incubated in goat anti-rabbit and anti-mouse biotinylated antibody (Vector Laboratories) at 1:300 in TBS for 2 h, and developed using the Elite ABC kit (Vector Laboratories) and diaminobenzidine (DAB) as a chromogen. Omission of the primary antibody eliminated all labeling. Sections were mounted on microscope slides and examined and photographed under an Olympus BX51 microscope equipped with AxioCam CCD camera (Zeiss) using the AxioVision imaging software (Zeiss).

### Multiple Label Immunofluorescence and Confocal Imaging

Immunofluorescence staining was carried out on free-floating sections. Antigen retrieval was performed with 2x Antigen Unmasking Solution Citrate Buffer pH 6.0 (Vector Laboratories, H-3300-250) in a steam cooker for 40 min at high temperature. The free-floating sections were left to cool for 15 min at RT. After washing the sections in deionized water, they were transferred to 1X TBS for 10 min. Sections were blocked for 1 h at RT in 1X TBS containing 5% bovine serum albumin, 2% Triton X-100, and 10% normal goat serum. Sections were incubated for 48 h at 4°C with specific primary antibodies GFAP (1:500, BioLegend, Cat# 829401), NeuN (1:300, EMD Millipore, Cat# ABN78), Iba1/AIF-1 (1:300, Cell Signaling Tech, Cat# 17198T), and GCPII (1:50, R&D Systems, Cat# MAB4234), followed by incubation overnight at 4°C with secondary antibodies (1:1,000, Alexa-Fluor conjugated, Invitrogen). Following incubation in secondary antibodies, they were incubated in 70% Ethanol with 0.3% Sudan Black B (MP Biomedicals, Cat# 4197-25-5), to decrease autofluorescence from lipofuscin, and counterstained with Hoechst (1:10,000, Thermo Fisher, Cat# H3570). The sections were mounted in ProLong Gold Antifade Mountant (Invitrogen, Cat# P36930).

Confocal images were acquired using a Leica TCS SP8 Gated STED 3X super resolution microscope, with the HC PL APO 100X/1.40 oil white objective (Leica) and HCX PL APO CS 63X/1.40 oil white objective (Leica). Emission filter bandwidths and sequential scanning acquisition were set up in order to avoid possible spectral overlap between fluorophores. Images were merged employing Fiji software.

### Biochemical Experiments

#### Western Analyses

Animals were anesthetized and rapidly decapitated. The brain was removed and the frontal block was rapidly dissected. Tissue was immediately frozen on dry ice for future analysis. Triton-soluble lysates were made as previously described (Datta et al., 2020, 2021). Briefly, tissue was rotor homogenized in a 1% Triton 100-X buffer with phosSTOP phosphatase inhibitor and cOmplete mini protease inhibitor. The triton-soluble fraction was collected following a 5 min 15,000xg centrifugation at room temperature. SDS-loading buffer with DTT was added to samples and they were boiled for 5 min prior to loading on a 4–20% Tris-glycine SDS-PAGE gel. Gels were transferred onto nitrocellulose membranes and blocked with 5% milk. Primary antibodies as described above were incubated at 4°C overnight. Blots were washed three times with PBS-T (0.1% Tween). Secondary antibodies were incubated for 1 h at room temperature and blots were again washed with PBS-T. Blots were imaged on a LiCor Odyssey Scanner and quantified with Image Studio Lite. Background was subtracted as the average intensity above and below the band of interest.

#### Statistical Analyses

Protein quantification was normalized by the GAPDH loading control within animals and normalized to the average young value across animals on the same blot. The distribution of protein levels was tested for normalcy using a D’Agostino and Pearson normality test. mGluR3 protein values were not normally distributed and thus were compared with a non-parametric Mann-Whitney test. GCPII protein values were normally distributed and there was no significant difference in variance between groups; thus, GCPII protein levels were compared using an independent *t*-test.

### 2-(3-Mercaptopropyl) Pentanedioic Acid Effects on Working Memory Performance

#### Cognitive Assessment

Rats were trained on the delayed alternation test of spatial working memory in a T-shaped maze. They were first adapted to handling and to eating treats (highly palatable miniature chocolate chips) on the maze prior to cognitive training. In this task, the rat was placed in the start box at the bottom of the “T.” When the gate was lifted, the rat proceeded down the stem of the maze to the choice point. On the first trial, rats were rewarded for entering either arm, but for each subsequent trial, were rewarded only if they chose the arm that they had not visited in the previous trial. Between trials, they were picked up and returned to the start box for a prescribed delay period. The choice point was cleaned with alcohol between each trial to remove olfactory clues (scent trails) often used by rodents to mark their previous locations. Successful performance of this task requires many operations carried out by the PFC: the rats must update and maintain the spatial information over the delay period for each trial, resist the distraction of being picked up and carried to the start box, and use response inhibition to overcome the tendency to repeat a rewarded action. Physiological recordings from monkey dlPFC have shown that despite the differing terms used to describe these seemingly distinct operations, they are all inter-related and reliant upon the fundamental process of whereby PFC neurons maintain neural representations across a delay period (Funahashi et al., 1993; Panichello and Buschman, 2021). In the current study, animals were tested daily for 10 trials/day, and many showed gradual, but steady, improvement in performance over the many months of the study. Thus, the delay period was adjusted for each rat, such that they were performing at a stable baseline of 60–80% before drug treatment (average near 70% correct), leaving room for either impairment or improvement in performance. The average delays for the young rats were 18.9 + 6.2 s (range of 0–55 s) with a median of 12.5 s, and for the aged rats, 13.3 + 5.3 s (range of 0–45 s) with a median of 6.3 s. The variation in delay lengths is typical of outbred rats (Morrison and Baxter, 2012); due to the variability in delays there was no significant difference between the age groups. Rats were tested by experimenters who were highly familiar with the normative behaviors of each individual animal, but blind to drug treatment conditions. Rats were observed for any potential differences in normative behavior (e.g., grooming, distracted sniffing), physical appearance, or physiological functioning (e.g., defecation/urination).

#### Drug Administration

The effects of acute administration of a dose of 2-MPPA on delayed alteration performance were compared to vehicle control. After achieving stable baseline performance (60–80% correct) for 2 consecutive days, rats were administered either vehicle (juice) or 2-MPPA (0.01, 0.1, 1.0, 10.0, or 20.0 mg/kg dissolved in vehicle; p.o.) in a counterbalanced order, with at least 1 week washout between treatments, with the exception that the 20 mg/kg dose was done last to ensure safety in aged animals. Drug solutions were administered 1 h prior to behavioral testing. All drug solutions were prepared fresh under sterile conditions immediately prior to use.

A second behavioral experiment examined the effects of an acute infusion of 2-MPPA directly into the prelimbic rat PFC (0.1 μg/0.5 μl per side vs. vehicle control) in a separate cohort of young adult rats (*n* = 8 male rats, ∼12 months old). For these experiments, cannula had been surgically implanted with tips aimed over the prelimbic PFC (AP: + 3.2 mm; ML: ± 0.75 mm; DV: –4.2 mm). Rats were adapted to the infusion procedure to minimize stress. Performance was assessed 10 min after the infusion by a researcher unaware of the drug treatment conditions.

#### Statistics

The effects of 2-MPPA on delayed alternation performance were assessed using a one way analysis of variance with repeated measures (within subjects design) using SPSS statistics software (IBM). As some aged rats died before they could be tested with all 6 doses, a repeated measures linear mixed model was used to allow inclusion of rats with missing data points in the statistical model. All aged rats completed the 1.0 mg/kg dose and thus this dosage was used for comparing variance in drug response between young and aged rats. The effects of infused 2-MPPA vs. vehicle into the PFC were analyzed with a paired *t*-test.

## Results

### Expression of Glutamate Carboxypeptidase II and mGluR3 in Young and Aged mPFC

#### Immunohistochemistry

**FIGURE 1:**
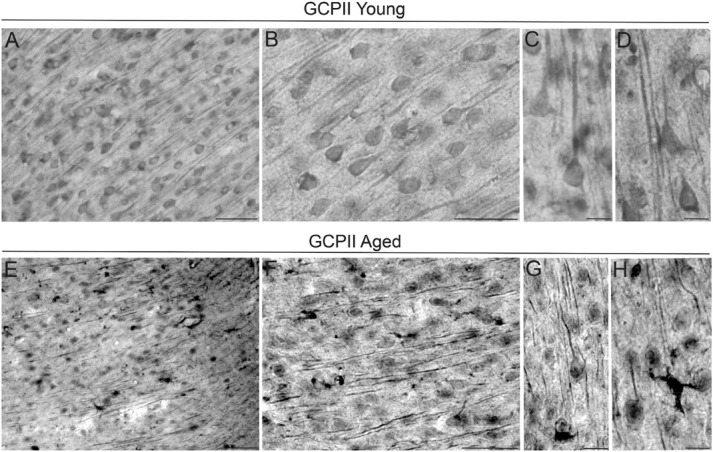
GCPII labeling by IHC in young and aged rat layer II/III PL PFC. **(A)** Immunolabeling for GCPII in young adult rat layer II/III PL mPFC revealing expression in multiple cell-types. Scale bar: 30 μm. **(B)** High-magnification micrograph for GCPII in young rat layer II/III PL mPFC revealing expression in glial cells and neurons. Pyramidal neurons in layer II/III were immunolabeled against GCPII, especially with staining found in the pyramidal perisomatic compartment, and diffuse labeling along apical dendrites. Scale bar: 30 μm. **(C,D)** High-resolution micrographs revealing GCPII immunolabeling in pyramidal cells in young rat layer II/III PL mPFC. Scale bars: 10 μm. **(E)** Expression of GCPII in aged rat layer II/III PL mPFC revealing aggregates of GCPII protein within intracellular compartments in multiple cell-types. **(F)** High-magnification micrograph for GCPII protein in aged rat layer II/III PL mPFC showing accumulation of GCPII protein within pyramidal cell apical dendrites and in non-neuronal cells. **(G)** High-resolution micrograph revealing GCPII immunolabeling in pyramidal cells and in apical dendrites traversing through the neuropil in aged rat layer II/III PL mPFC. Scale bar: 10 μm. **(H)** GCPII labeling was also observed in non-neuronal glial cells, extending delicate processes in the neuropil. Scale bar: 10 μm.

The localization of GCPII immunoreactivity in the mPFC PL layer II/III of young adult and aged rats was examined using immunohistochemistry. In general, GCPII-IR was abundant in both young and aged rats, with labeling in multiple cell-types and punctate labeling in neuropil (Figure 1). In young and aged rats, there was GCPII labeling in neurons with distinct pyramidal cell-like morphological features, as well as labeling in smaller glial-like cells. In young rats, GCPII labeling was more diffuse within intracellular compartments (Figures 1A,B), while in aged rats, GCPII immunoreactivity was more aggregated within subcellular compartments (e.g., pyramidal cell dendritic shafts; Figures 1C,D). There was also dense labeling of cells with a morphological phenotype consistent with putative reactive astrocytes (Figures 1C,D, highlit in Figure 1E). Given that GCPII mRNA is known to be limited to astrocytes (Berger et al., 1999), the prominent labeling of GCPII protein on neurons is a novel and important finding.

**FIGURE 2:**
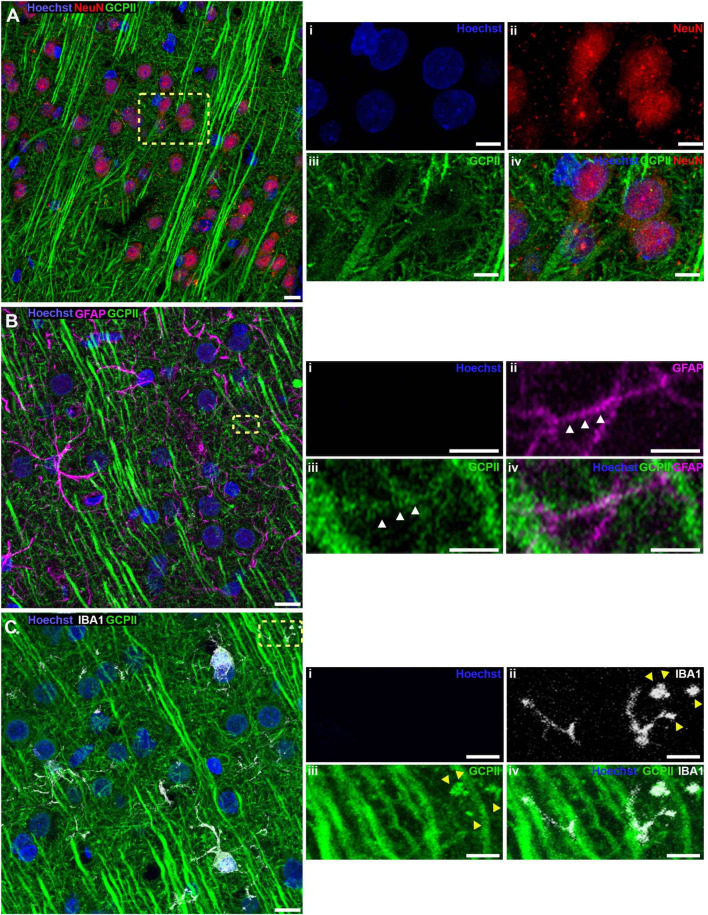
Neuronal, astrocytic, and microglial expression of GCPII in layer II/III of rat PFC by IF. **(A)** Representative confocal images of aged rat layer II/III PL mPFC stained for NeuN (*red)*, GCPII *(green)*, and nuclei were counterstained with Hoechst (*blue)*. Images taken at 63X magnification. **(Ai–iv)** Show magnified images by channel of the region outlined by the yellow dashed box on the composite image. Note the prominent presence of GCPII in the parallel apical dendrites projecting toward the pia and NeuN positive soma of pyramidal neurons, as identified by the white triangles. Scale bars: **(A)** 10 μ*m*, **(Ai-iv)** 5 μ*m*. **(B)** Representative confocal images of aged rat layer II/III PL mPFC stained for GFAP *(magenta)*, GCPII *(green)*, and nuclei were counterstained with Hoechst *(blue)*. Images taken at 100X magnification. **(Bi–iv)** Show magnified images by channel of the region outlined by the yellow dashed box on the composite image. Note the delicate labeling of a GFAP positive astrocytic process with GCPII demarcated by white triangles. Scale bars: **(B)** 10 μm, **(Bi–iv)** 2.5 μm. **(C)** Representative confocal images of aged rat layer II/III PL mPFC stained for Iba1 *(gray)*, GCPII *(green)*, and nuclei were counterstained with Hoechst *(blue)*. Images taken at 100X magnification. **(Ci–iv)** Show magnified images by channel of the region outlined by the yellow dashed box on the composite image. Note the Iba1 positive microglial process labeled with GCPI demarcated by yellow triangles. It is possible that this labeling reflects phagocytosed fragments of a GCPII-expressing neuron. Scale bars: **(C)** 10 μm, **(Ci–iv)** 2.5 μm.

The localization of GCPII immunoreactivity in PL mPFC layer II/III was further corroborated using multi-label immunofluorescence to identify expression in neurons vs. astrocytes vs. microglia in aged rat PL mPFC (Figure 2). There was robust GCPII labeling of neurons, identified by NeuN positive soma and distinct pyramidal cell-like morphological features. GCPII was expressed throughout the apical dendrites as well as the neuronal soma, excluding the nucleus (Figure 2A). In order to identify the non-neuronal cell-types that share GCPII expression, we co-stained for an astrocytic marker, GFAP, or a microglial marker, Iba1. There was delicate colocalization of both Iba1 and GFAP with GCPII, indicating that GCPII protein is subtly expressed by these two glial cell types (Figures 2B,C). GFAP and GCPII co-expression were especially evident in more distal astroglial processes, the sites of likely synaptic-astroglial interactions. Altogether, these data are consistent with previous findings in prostate where GCPII protein may be synthesized and released by one cell type (e.g., astrocytes), and taken up by others (e.g., neurons).

**FIGURE 3:**
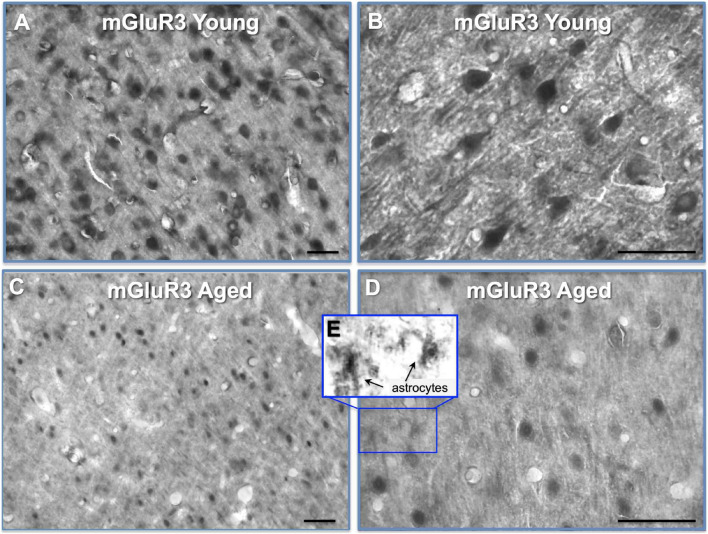
mGluR3 labeling by IHC in young and aged rat layer II/III PL PFC. **(A)** Immunolabeling for mGluR3 in young rat layer II/III PL mPFC revealing high density of mGluR3 expression in multiple cell-types. **(B)** High-magnification micrograph showing mGluR3 expression in young rat layer II/III PL mPFC in excitatory pyramidal cells, and smaller putative astrocyte-like cells. The neuropil is also characterized by punctate-like labeling for mGluR3. **(C)** Expression of mGluR3 in aged rat layer II/III PL mPFC with a marked decrement in the density of labeling across cell-types. **(D,E)** High-magnification micrograph showing mGluR3 expression in aged rat layer II/III PL mPFC with sparser labeling, and decreased labeling in dendritic arbors, as well as possible labeling of reactive astrocytes, magnified in insert **(E)**. Scale bars: 50 μm.

A previous study had shown reductions in mGluR3 protein and mRNA in rat PL mPFC with advanced age (Hernandez et al., 2018). Thus, we localized mGluR3 protein labeling in young vs. aged rat PL mPFC (Figure 3). In young rat PL mPFC, mGuR3 labeling was prominent on neurons with pyramidal-like phenotypes, as well as on smaller cells and neuropil labeling that likely included astrocytic processes (Figures 3A,B), consistent with the known locations of mGluR3 in the rodent. Comparisons between the young and aged immunohistochemical labeling of PL mPFC showed evidence of a general reduction in mGluR3 neuronal labeling with age (Figures 3C,D), consistent with Hernandez et al., 2018. There was also evidence of mGluR3 labeling of likely astrocytes in the aged tissue, including some with extensive branching and stellate-like appearance, suggesting a “reactive-like” phenotype (Figures 3D,E), as has been seen in human cell cultures (Aronica et al., 2003; Clarke et al., 2018).

#### Biochemistry

**FIGURE 4:**
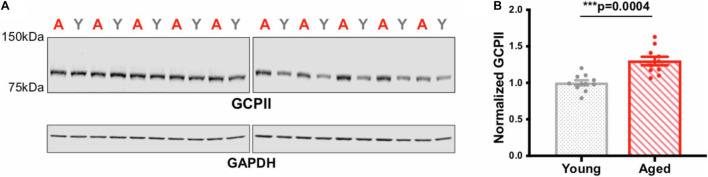
GCPII levels in young vs. aged rat frontal cortex. **(A)** Rat frontal cortex tissue (18 μg of protein loaded per well) was immunoblotted for GCPII and GAPDH. Each lane represents a single animal and is labeled either young (gray Y) or aged (red A). **(B)** Quantification of GCPII normalized by GAPDH grouped by age. Young animals (*n* = 10) and aged animals (*n* = 10) are compared via an independent *t*-test (***p = 0.0004). SEM is plotted for each group.

Levels of protein expression were quantified using western blotting of the frontal block, which included PL mPFC but also surrounding tissues. Western assays of GCPII expression in young vs. aged rat frontal cortex showed a significant increase with age (^∗∗∗^*p* = 0.0004; Figure 4; all blots in Supplementary Figure 1). These biochemical results are consistent with the denser IHC labeling in aged rat mPFC.

**FIGURE 5:**
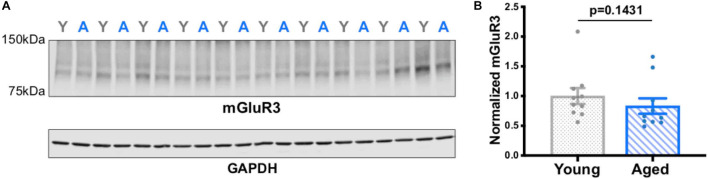
mGluR3 levels in young vs. aged rat frontal cortex. **(A)** Rat frontal cortical tissue (15 μg of protein loaded per well) was immunoblotted for mGluR3 and GAPDH. Each lane represents a single animal and is labeled either young (gray Y) or aged (blue A). **(B)** Quantification of mGluR3 normalized by GAPDH grouped by age. The young animals (*n* = 10) and aged animals (*n* = 10) were compared via a Mann-Whitney test (*p* = 0.1431). SEM is plotted for each group.

Western analyses of the frontal block showed only a trend level reduction in mGluR3 expression with age (Figure 5; all blots in Supplementary Figure 1). These mixed results may have been due to the assay capturing mGluR3 levels in the entire frontal block, whereas previous results showed selective, age-related reductions in mGluR3 expression only in the PL mPFC (Hernandez et al., 2018).

### Effects of Glutamate Carboxypeptidase II Inhibition on Delayed Alternation Performance

**FIGURE 6:**
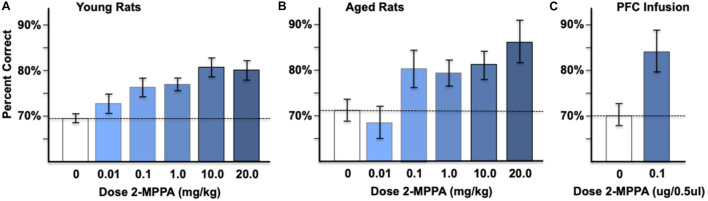
Administration of the GCPII inhibitor, 2-MPPA, improves performance on the delayed alternation test of spatial working memory. **(A)** The effects of oral administration of 2-MPPA on working memory performance in young adult rats (*n* = 9). **(B)** The effects of oral administration of 2-MPPA on working memory performance in aged (*n* = 11) rats. 2-MPPA produced significant, dose-related improvements in percent correct on the spatial delayed alternation task in a T maze in both age groups (see text). **(C)** Infusion of 2-MPPA directly into the adult rat PFC (*n* = 8) significantly improved working memory performance. Results are expressed as the mean ± SEM.

Systemic administration of the GCPII inhibitor, 2-MPPA: The effects of GCPII inhibition on delayed alternation performance were tested in young adult and aged rats using oral administration of 2-MPPA, the most brain penetrant GCPII inhibitor currently available (Vornov et al., 2013). Rats performed the delayed alternation task in a T maze, a test of spatial working memory that is dependent on the integrity of the mPFC. Administration of 2-MPPA (0.01–20 mg/kg p.o.,1 h before testing) compared to vehicle control produced a dose-related, significant improvement in working memory performance in both young adult rats (Figure 6A) and aged rats (Figure 6B). In young adult rats, there was a significant effect of 2-MPPA dosage [*F*(5, 40) = 4.73, *p* = 0.002], with a linear dose/response [*F*(1, 8) = 26.5, *p* = 0.001]. *Post hoc* tests showed significant improvements at the 1.0 mg/kg (*p* = 0.032) and 10.0 mg/kg (*p* = 0.011) doses, and a trend for improvement with the 20 mg/kg dose (*p* = 0.072).

Systemic administration of 2-MPPA to aged rats also improved delayed alternation performance. As 4 aged rats died before receiving all doses (particularly the 20 mg//kg dose), a repeated measures linear mixed model was used to allow inclusion of rats with missing data points. In aged adult rats, there was a significant effect of 2-MPPA dosage [*F*(5, 50) = 4.7, *p* = 0.001] with a linear dose/response [*F*(1, 54) = 8.7, *p* = 0.005]. *Post hoc* tests showed significant improvements at the 0.1 mg/kg (*p* = 0.036), 1.0 mg/kg (*p* = 0.047), 10.0 mg/kg (*p* = 0.027), and 20.0 mg/kg (*p* = 0.005) doses. Importantly, there were no apparent side effects at any dose of 2-MPPA in young or aged rats, consistent with the excellent side effect profiles of GCPII inhibitors reported previously (Zhang et al., 2006).

As all young and aged rats received the 1.0 mg/kg dose, a more detailed qualitative analysis of this dose was performed. 1.0 mg/kg 2-MPPA significantly improved accuracy in both the young (*p* = 0.002) and aged (*p* = 0.047) rats compared to vehicle control. However, performance was more variable in the aged rats, for both vehicle and drug performance, as reflected in the larger SEMs. For example, the aged rats had more variable performance under vehicle control conditions (*F*-test, *p* = 0.023) and following the 1.0 mg/kg dose, where performance ranged from 70 to 80% correct for young rats (a 7.6% + 1.8% improvement from their vehicle control), but 70–100% for aged rats (a 7.9% + 3.5% improvement from their vehicle control), with significantly greater variability (i.e., larger SEM) in the aged rats (*F*-test, *p* = 0.0396). Response times following the 1.0 mg/kg dose were not significantly different from vehicle control in either young or aged rats (young rats vehicle-drug averaged 11.7 + 22 s, *p* = 0.59; aged rats averaged –13.5 + 15 s, *p* = 0.38).

Intra-PFC infusions of the GCPII inhibitor, 2-MPPA: Infusion of 2-MPPA (0.1 μg/0.5 μl/side) directly into the rat PL mPFC significantly improved delayed alternation performance compared to infusion of vehicle control (*p* = 0.014, Tdep, *n* = 8; Figure 6C). These results are consistent with at least some of the beneficial effects of systemic treatment arising from drug actions within the PFC.

## Discussion

There is renewed interest in the GCPII-NAAG-mGluR3 signaling cascade given the strong relationship between this pathway and cognitive deficits in humans. The current study aimed to analyze the role of GCPII in the aging rat mPFC, a model frequently used to assess molecular mechanisms related to cognitive disorders. We found substantial expression of GCPII in rat mPFC, including an age-related increase in GCPII levels, which may contribute to cognitive deficits by reducing beneficial NAAG actions at mGluR3. Inhibition of GCPII with either systemic or local 2-MPPA administration significantly improved working memory performance, consistent with a detrimental effect of endogenous GCPII signaling on cognition. As GCPII expression is increased with inflammation (Vornov et al., 2016; Zhang Z. et al., 2016), these data may help to explain the cognitive deficits that accompany a large number of inflammatory disorders (Rahn et al., 2012), and provide a potential avenue for therapeutic intervention.

### Neuronal as Well as Glial Expression of Glutamate Carboxypeptidase II Protein

Previous studies have emphasized the localization of GCPII mRNA in astrocytes (Berger et al., 1999), and GCPII protein expression in astrocytes, microglia, and neuropil (Slusher et al., 1992; Berger et al., 1999; Marmiroli et al., 2012; Zhang Z. et al., 2016; Arteaga Cabeza et al., 2021). The current data confirmed GCPII expression in astrocytes and microglia, but additionally observed extensive labeling of neurons with a pyramidal-like phenotype. The lack of neuronal GCPII labeling in the original studies may be due to the use of less sensitive techniques such as *in situ* hybridization and assays of DNA levels (Ghose et al., 2009), often carried out in cell cultures, rather than visualizing GCPII protein *ex vivo.* Although studies using monoclonal antibodies *ex vivo* have also reported primarily glial labeling (reviewed in Marmiroli et al., 2012). The current study used procedures that permeabilize neuronal membranes, which may have allowed superior antibody access into neurons. Neuronal GCPII labeling has also been reported in mouse hippocampus (Huang et al., 2004), and in the peripheral nervous system in dorsal root ganglia and sciatic nerves (Carozzi et al., 2008), consistent with the current results. Immunoelectron microscopy data also show GCPII in the spines and dendrites of aged rhesus monkey dorsolateral PFC (Datta and Arnsten, unpublished), consistent with the current interpretation. It is known that GCPII is endocytosed in prostate epithelium, where it is synthesized in epithelial cells and taken up by neighboring cells (Bařinka et al., 2012), and it is possible that a similar process occurs in PFC, where GCPII is synthesized in astrocytes and then taken up into neighboring neurons (especially into dendrites), and that this process may vary based on environmental conditions.

In particular, the levels of GCPII expression may differ between studies based on inflammatory state, as GCPII expression increases with inflammation, and can be seen in both reactive astrocytes (Ha et al., 2016), and reactive microglia (Zhang Z. et al., 2016; Arteaga Cabeza et al., 2021). These findings are in line with the current data showing GCPII labeling of cells with potentially reactive glia-like profiles in the aged rat mPFC, consistent with increased inflammatory signaling (e.g., complement cascade pathways) in aged PFC (Datta et al., 2020). Increased inflammation may also increase GCPII uptake into neurons. For example, GCPII labeling of neurons was increased in both cell cultures exposed to hypoxia, and in rat cortical neurons following ischemic injury (Zhang W. et al., 2016). Thus, neuronal GCPII labeling may be related to the degree of exposure to inflammatory conditions. Although the role of inflammation in the aged PFC was not directly manipulated in the current study, this would be a relevant area for future research, e.g., to determine whether GCPII expression in dendrites increased with exposure to inflammatory events. The concentration of GCPII in pyramidal cell dendrites is also consistent with a recent study demonstrating NAAG expression within hippocampal dendrites, where it can be released to engage mGluR3 (Nordengen et al., 2020). Future research could examine the role(s) of GCPII and NAAG expression in neurons, and whether their uptake and/or release alters neuronal firing.

### Age-Related Changes in Glutamate Carboxypeptidase II-mGluR3 Signaling

The current data suggest that NAAG-mGluR3 signaling is decreased with advancing age in rat mPFC due to age-related increases in GCPII expression, and possible mGluR3 reduction. Reduced mGluR3 IHC labeling in aged PL mPFC was seen in the current study, consistent with previous data showing age-related reductions in mGluR3 protein and mRNA in the PL mPFC, but not nearby subregions (Hernandez et al., 2018). The specificity to the prelimbic region may explain why the current study found only a trend-level reduction in mGluR3 protein levels in frontal cortex as measured by western blotting, as the block dissected for biochemical analysis included subregions outside of the PL mPFC.

Both IHC and biochemical measures showed increased GCPII with age, including glia with morphological characteristics suggestive of a “reactive” phenotype in aged mPFC. Increased GCPII expression likely corresponds with decreased NAAG-mGluR3 signaling, which may contribute to cognitive deficits, vulnerability to excitotoxicity, calcium dysregulation, and further inflammation. It is likely that the increase in GCPII expression in the aged rat PFC is a result of increased inflammation with age, as GCPII expression is induced by inflammation in young brain (Zhang Z. et al., 2016). GCPII inhibitors have beneficial effects in a number of inflammatory and/or excitotoxic conditions (Carpenter et al., 2003; Berent-Spillson et al., 2004; Neale et al., 2005; Zhang et al., 2006; Rahn et al., 2012; Wozniak et al., 2012; Nonaka et al., 2017), including improving learning and memory in a mouse model of multiple sclerosis (Rahn et al., 2012), while genetic knockout of GCPII protects cortical and hippocampal neurons against traumatic brain injury (Cao et al., 2015, 2016).

### Possible Mechanisms Underlying Glutamate Carboxypeptidase II Inhibition Improvement in Working Memory

The current study found improved performance of the delayed alternation task following either systemic or local mPFC administration of the GCPII inhibitor, 2-MPPA, which unlike many GCPII inhibitors, is able to cross the blood-brain barrier (BBB). Systemic administration improved performance in both young adult and aged rats, consistent with GCPII being expressed in young as well as aged animals, albeit at higher levels in the aged. This suggests that even moderate levels of GCPII expression are harmful to PFC cognitive abilities. The infusion of 2-MPPA directly into the PL mPFC also improved performance of the task, suggesting that at least some of the enhancing effects of GCPII inhibition occurred within the mPFC. These are the first data showing that GCPII inhibition can improve mPFC function. mGluR3 signaling can also enhance hippocampal associative learning (Dogra et al., 2021), and thus there may be other aspects of cognitive networks that are improved by systemic treatment with a GCPII inhibitor (see Neale and Olszewski, 2019) for a recent review of mGluR3 and cognition.

**FIGURE 7:**
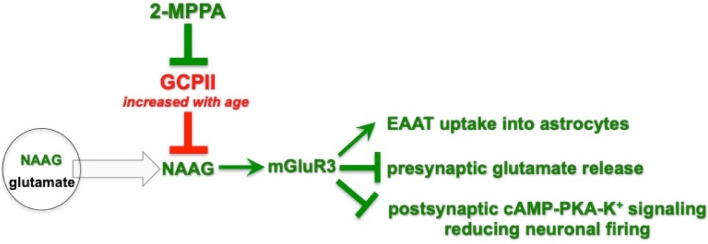
Schematic diagram of 2-MPPA inhibition of GCPII-NAAG-mGluR3 signaling, and some of the mechanisms in mPFC that may influence working memory performance. NAAG is co-released with glutamate from presynaptic vesicles, but unlike glutamate, is selective for mGluR3. GCPII catabolizes NAAG, while 2-MPPA inhibits GCPII activity, increasing NAAG stimulation of mGluR3. Acute increases in NAAG-mGluR3 signaling with 2-MPPA administration may improve working memory performance via a number of mechanisms, including limiting the spread of glutamate actions via increased glutamate uptake into astroctyes via excitatory amino acid transporters (EEATs) and reduced glutamate release from axons, and/or strengthening of network connections via post-synaptic mGluR3 inhibition of cAMP-K^+^ channel signaling on spines.

There are a number of mechanisms by which reduced GCPII activity, and increased NAAG-mGluR3 stimulation, might benefit working memory performance in PFC (Figure 7). Recent data from studies of the macaque have shown that NAAG-mGluR3 signaling has unusual actions in layer III of the primate dlPFC, where recurrent cortical-cortical connections reside (Jin et al., 2018). Stimulation of mGluR3 by NAAG enhances the neuronal firing underlying working memory via post-synaptic mGluR3 on spines, which inhibit cAMP-PKA opening of K^+^ channels to strengthen synaptic connectivity (Jin et al., 2018). mGluR3 can also be found on a subset of spines in layer III of rat PL mPFC (Woo, Datta and Arnsten, unpublished). As this layer is also a focus of inputs from the rhinal cortices, MD thalamus and hippocampus (Jay and Witter, 1991; Parent et al., 2010; Collins et al., 2018; Hwang et al., 2018; Canetta et al., 2020), if post-synaptic mGluR3 reside on these connections, they may similarly strengthen working memory-related neuronal connections in rats (Spellman et al., 2015; Bolkan et al., 2017; Schmitt et al., 2017; Halassa and Sherman, 2019). GCPII inhibition may also be beneficial by increasing mGluR3 actions on astrocytes and on axons (Figure 7), where enhanced uptake of glutamate and reduced volume release may reduce glutamate spread and reduce “noise.” Studies using non-selective mGluR2/3 agents also support a role in working memory, where agonists increase the expression of phosphorylated NMDAR in rat mPFC, and ameliorate dysfunction caused by NMDAR blockade (Xi et al., 2011), while antagonist infusion impaired working memory performance (Hernandez et al., 2018).

### Relevance to Potential Treatments

mGluR3 and GCPII expression are increasingly relevant to human cognitive abilities, and to the etiology and treatment of mental disorders. GRM3 is a replicated GWAS risk gene for schizophrenia (Saini et al., 2017), and variations in *FOLH1* genotype that increase GCPII expression and lower NAAG-mGluR3 signaling are associated with lower IQ scores (Zink et al., 2020). Lower NAAG levels are also associated with cognitive deficits in patients with multiple sclerosis (Rahn et al., 2012). These results in humans are consistent with data from monkeys, where mGluR3 signaling on spines strengthens dlPFC network connectivity, and GCPII inhibition enhances working memory-related neuronal firing (Jin et al., 2018). The current finding that systemic administration of a BBB permeable GCPII inhibitor can improve working memory performance in rats, with no evidence of side effects, encourages the further development of this mechanism for treating cognitive disorders in humans. Administration of GCPII inhibitors to rodents has previously been shown to improve performance of tests of learning and memory that depend on the hippocampus (Janczura et al., 2013), including in mouse models of AD and substance abuse (Olszewski et al., 2017) or of multiple sclerosis (Rahn et al., 2012; Hollinger et al., 2016). Thus, these agents may have broad applicability for treating impairments in both learning and working memory, and may be especially useful in conditions such as aging associated with neuroinflammation.

## Data Availability Statement

The raw data supporting the conclusions of this article will be made available by the authors, without undue reservation.

## Ethics Statement

The animal study was reviewed and approved by the Yale IACUC.

## Author Contributions

DD, SL, BS, AN, and AA designed the study. BS supplied drug. DD, SL, EW, NA, AE, and ML collected and analyzed the data. AM performed statistical analysis and interpretation, and made critical revisions to the manuscript. AA wrote the first draft, with all authors contributing to the final version.

## Conflict of Interest

The authors declare that the research was conducted in the absence of any commercial or financial relationships that could be construed as a potential conflict of interest.

## Publisher’s Note

All claims expressed in this article are solely those of the authors and do not necessarily represent those of their affiliated organizations, or those of the publisher, the editors and the reviewers. Any product that may be evaluated in this article, or claim that may be made by its manufacturer, is not guaranteed or endorsed by the publisher.

## Supplementary Material

Supplementary Figure 1 Full western blot membranes. **(A)** Image of the full nitrocellulose membrane, rather than just the region of interest, from Figure 4. Rat frontal cortex tissue (18 mg of protein loaded per well) was immunoblotted for GCPII and GAPDH. Each lane represents a single animal and is labeled either young (gray Y) or aged (red A). **(B)** Image of the full nitrocellulose membrane, rather than just the region of interest, from Figure 5. Rat frontal cortical tissue (15 mg of protein loaded per well) was immunoblotted for mGluR3 and GAPDH. Each lane represents a single animal and is labeled either young (gray Y) or aged (blue A).
