# Supplementary figures and images for: Glutamate Carboxypeptidase II in Aging Rat Prefrontal Cortex Impairs Working Memory Performance
Source: Front Aging Neurosci. 2021 Nov 15;13:760270. doi: 10.3389/fnagi.2021.760270 (PMC8634091; doi:10.3389/fnagi.2021.760270)

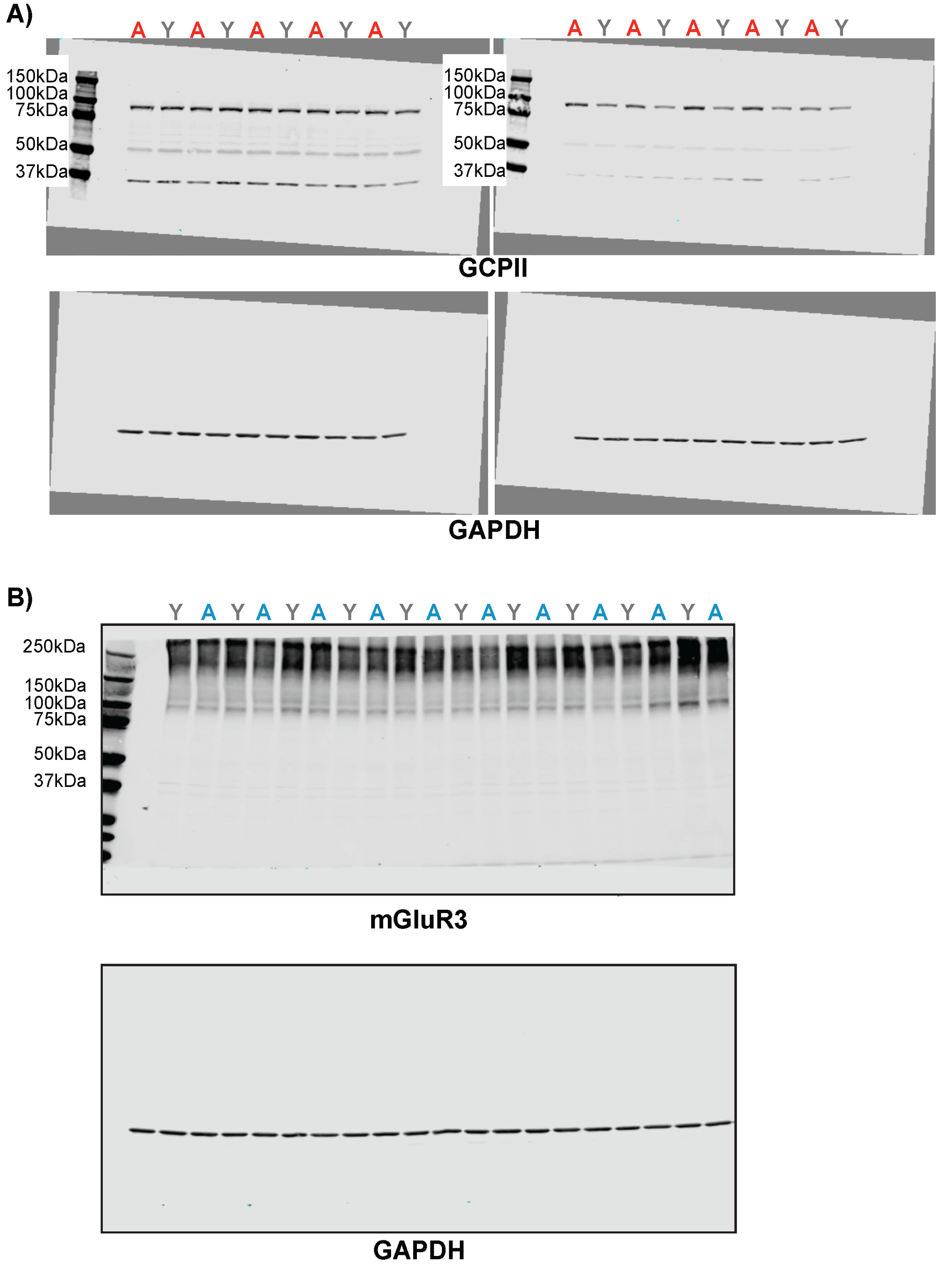

Supplement: Supplementary Figure 1 — Full western blot membranes. (A) Image of the full nitrocellulose membrane, rather than just the region of interest, from Figure 4. Rat frontal cortex tissue (18 mg of protein loaded per well) was immunoblotted for GCPII and GAPDH. Each lane represents a single animal and is labeled either young (gray Y) or aged (red A). (B) Image of the full nitrocellulose membrane, rather than just the region of interest, from Figure 5. Rat frontal cortical tissue (15 mg of protein loaded per well) was immunoblotted for mGluR3 and GAPDH. Each lane represents a single animal and is labeled either young (gray Y) or aged (blue A). [file Image_1.TIFF]
